# WNT‐5A triggers Cdc42 activation leading to an ERK1/2 dependent decrease in MMP9 activity and invasive migration of breast cancer cells

**DOI:** 10.1016/j.molonc.2013.04.005

**Published:** 2013-04-28

**Authors:** Chandra Prakash Prasad, Shivendra Kumar Chaurasiya, Lena Axelsson, Tommy Andersson

**Affiliations:** ^1^ Cell and Experimental Pathology, Department of Laboratory Medicine, Lund University, Clinical Research Centre, Skåne University Hospital, SE-20502 Malmö, Sweden

**Keywords:** WNT-5A, Breast cancer, Cdc42, ERK1/2, MMP9

## Abstract

An important role for WNT‐5A is implicated in a variety of tumors, including breast carcinoma. We previously showed that WNT‐5A signaling inhibits migration and metastasis of breast cancer cells, and that patients with primary breast cancer in which WNT‐5A was expressed have a better prognosis. Despite the fact that RhoGTPase Cdc42 is commonly associated with increased cell migration, we here show that recombinant WNT‐5A activates the Cdc42 in breast cancer cells (lines MDA‐MB468 and MDA‐MB231) in a time‐dependent manner. Activation of Cdc42 was also observed in MDA‐MB468 cells that were stably transfected with a WNT‐5A plasmid (MDA‐MB468‐5A). In all situations, increased Cdc42 activity was accompanied by decreased migration and invasion of the breast cancer cells. To explore these findings further we also investigated the effect of WNT‐5A signaling on ERK1/2 activity. Apart from an initial Ca2+‐dependent rWNT‐5A‐induced activation of ERK1/2, Cdc42 activity was inversely correlated with ERK1/2 activity in both rWNT‐5A‐stimulated parental MDA‐MB468 and MDA‐MB468‐5A cells. We also demonstrated increased ERK1/2 activity in MDA‐MB468‐5A cells following siRNA knockdown of Cdc42. Consistent with these results, breast cancer cells transfected with constitutively active Cdc42 exhibited reduced ERK1/2 activity, migration and invasion, whereas cells transfected with dominant negative Cdc42 had increased ERK1/2 activity in response to rWNT‐5A. To gain information on how ERK1/2 can mediate its effect on breast cancer cell migration and invasion, we next investigated and demonstrated that WNT‐5A signaling and constitutively active Cdc42 both decreased matrix metalloproteinase 9 (MMP9) activity. These data indicate an essential role of Cdc42 and ERK1/2 signaling and MMP9 activity in WNT‐5A‐impaired breast cancer cells.

## Highlights

- A controversy exists regarding the role of WNT‐5A in breast cancer cell migration.
- Surprisingly, WNT‐5A‐induced Cdc42 activation leads to decreased breast cancer cell migration and invasion.
- WNT‐5A triggers cross‐talk between Cdc42 and ERK1/2 thereby regulating MMP9 activity.
- WNT‐5A mediates a tumor suppressor effect on breast cancer cells.

**Abbreviations**
Cdc42: cell division control protein 42 homolog
MMP: matrix metalloproteinase
PLB: phosphorylation lysis buffer
rWNT-5A: recombinant WNT-5A
rMMP9: recombinant MMP9

## 1 Introduction

WNTs constitute a large family of secreted cysteine‐rich proteins that, by activating multiple intercellular signaling pathways (Cadigan and Nusse, 1997; Dale, 1998; Kikuchi et al., 2011; Miller et al., 1999), are vital in the regulation of cellular processes such as proliferation, differentiation, apoptosis, survival, migration, and polarity. Signaling is initiated when a WNT ligand binds to a specific receptor or a receptor‐co‐receptor complex, including Frizzled, ROR1/2, RYK, and/or low‐density lipoprotein receptor‐related protein 5/6 (LRP5/6) resulting in the initiation of diverse cellular processes (Lai et al., 2009; Nusse, 2008; Schulte, 2010). The WNT ligands can be divided into three distinct classes based on differences in their ability to transform mouse mammary cells (Shimizu et al., 1997); the highly transforming group, including WNT‐1, WNT‐3A, WNT‐7A, WNT‐2, and WNT‐5B; a non‐transforming group, including WNT‐4, WNT‐5A, and WNT‐6; and an intermediate transforming group, including WNT‐7B. This categorization does not necessarily reflect the specific signaling pathways that WNTs initiate (Lai et al., 2009; MacDonald et al., 2009; Moon et al., 2004), and an extensive signaling crosstalk exists between WNT ligands with different transforming capacities (Buechling and Boutros, 2011).

Experiments performed on C57MG cells (an epithelial cell line derived from normal mouse mammary tissue) showed that unlike WNT‐1, WNT‐3A and WNT‐7A, WNT‐5A failed to induce cell transformation in these cells (Wong et al., 1994), and that the expression of exogenous WNT genes (e.g. WNT‐1 and WNT‐2) lead to a downregulation of WNT‐5A and a more efficient cell transformation (Olson and Papkoff, 1994). Subsequent experiments revealed that ectopic expression of WNT‐5A in tumorigenic uroepithelial cells resulted in suppressed anchorage–independent growth and reduced tumorigenicity when inoculating the cells into athymic nude mice (Olson et al., 1997). In support of these findings, our group has shown that WNT‐5A can reverse the effect on WNT‐3A‐induced Topflash reporter activity in A2058 melanoma cells (Ekstrom et al., 2011).

The role of WNT‐5A in tumor initiation and progression is complex as differences in cellular constitutions such as receptor repertoire can trigger diverse signaling pathways. This is probably why WNT‐5A acts as a tumor suppressor in breast cancer, colon cancer, thyroid cancer, liver cancer, and lymphoid malignancies (Dejmek et al., 2005a; Jiang et al., 2013; Jonsson et al., 2002; Kremenevskaja et al., 2005; Liu et al., 2008; Roman‐Gomez et al., 2007) but promotes tumor progression in malignant melanoma and gastric cancer (Da Forno et al., 2008; Kurayoshi et al., 2006; Weeraratna et al., 2002).

In primary invasive breast cancer tissue, we previously reported that low expression of WNT‐5A protein is associated with a higher histological grade of tumors and earlier disease recurrence in patients (Jonsson et al., 2002), reflecting a more rapid development of distant metastases. In a different cohort of patients, low expression of WNT‐5A protein in breast carcinomas was associated with reduced overall survival (Dejmek et al., 2005b). These findings indicate a suppressive role of the WNT‐5A protein in breast cancer progression. Translational regulation of WNT‐5A mRNA (Clark et al., 1993; Dejmek et al., 2005b; Leandersson et al., 2006; Sheehy et al., 2010) indicates that the WNT‐5A mRNA level in human breast cancer tissue differs from the WNT‐5A protein level in the same tissue sample. Therefore, it is important to be cautious when correlating WNT‐5A mRNA levels to clinical outcome of patients.

These findings point to the importance of identifying the molecular mechanisms responsible for the tumor suppressing effect of the WNT‐5A protein in breast cancer. Its expression in clinical breast cancer samples was not associated with the expression of the proliferation markers Ki‐67, Cyclin D1, or Rb, suggesting that WNT‐5A does not inhibit the proliferation of these tumors (Dejmek et al., 2005b). This conclusion is supported by *in vitro* studies on 4T1 breast cancer cells, in which WNT‐5A had no effect on proliferation and apoptosis (Safholm et al., 2008).

Other *in vitro* studies of breast cancer cells have led to the suggestion that WNT‐5A reduces the metastatic capability of invasive breast cancer by enhancing adhesion and decreasing migration (Dejmek et al., 2003; Jiang et al., 2013; Jonsson and Andersson, 2001; Medrek et al., 2009; Roarty and Serra, 2007; Safholm et al., 2006). However, there are some reports that question the ability of WNT‐5A to inhibit the migration of breast cancer cells and suggest that WNT‐5A increases migration of breast cancer cells (Pukrop et al., 2006; Zhu et al., 2012). The proposal that WNT‐5A increases breast cancer cell migration is compatible with its capacity to increase Cdc42 activity in fibroblasts (Schlessinger et al., 2007) and non‐tumorigenic breast epithelial cells (Dejmek et al., 2006), since Cdc42 activity is commonly associated with increased cell migration (Fritz et al., 1999; Karlsson et al., 2009; Tang et al., 2008).

To explore these discrepancies, we investigated the precise role of Cdc42 in WNT‐5A‐mediated signaling in breast cancer cells. We employed two different experimental strategies. First, we evaluated the primary effects of WNT‐5A by stimulating the cells with rWNT‐5A. Second, we established a breast cancer cell line with stable expression of the WNT‐5A protein to study its long‐term effects. We also incorporated Matrigel invasion studies, as it is believed that this approach better reflects the *in‐vivo* situation (Kleinman and Martin, 2005; Lee et al., 2008), by providing a more appropriate extracellular matrix environment.

In this study, we report that WNT‐5A‐mediated activation of Cdc42 leads to decreased breast cancer cell migration and invasion via reduced ERK1/2 and MMP‐9 activities.

## 2 Materials and methods

### 2.1 Cell lines

Characterized batches of the human mammary carcinoma cell lines MDA‐MB468 (Lot No. 58483213) and MDA‐MB231 (Lot No. 58629943) were purchased and delivered directly from ATCC in 2011. The cells were regularly tested for their absence of mycoplasma (EZ‐PCR kit by Biological Industries, Haemek, Israel). The cells were grown in DMEM D5796 media supplemented with 10% FBS, 5 U/mL penicillin, 0.5 U/mL streptomycin, and 2 mM glutamine.

### 2.2 Plasmid and siRNA transfections

MDA‐MB468 cells stably transfected with WNT‐5A were obtained by plating 7.5 × 10^5^ cells in 100‐mm‐diameter cell‐culture dishes prior to lipofectamine transfection with either pcDNA3.1(+)‐WNT‐5A plasmid or a pcDNA3.1 empty vector (Ying et al., 2008). Cells were transfected with 15 μg (12 μg in the case of empty vector) of plasmid DNA complex with 30 μL Lipofectamine 2000 transfection reagent and incubated with the transfection complex for 6 h. Cells were then gently rinsed with growth medium and fresh complete growth medium (DMEM supplemented with 10% FBS and 700 μg/mL Geneticin [G418]) was added to the dishes 24 h after transfection for selection of stable transfectants. Forty‐eight hours after transfection, growth medium supplemented with 800 μg/mL (as determined by kill curve) G418 was added for further selection of stable transfectants. The stable MDA‐MB468 cells expressing WNT‐5A (MDA‐MB468‐5A) were maintained in DMEM supplemented with 10% FBS and 700 μg/mL G418. Prior to assays (migration, invasion, and Western blot analysis) the cells were washed with PBS and grown for 48 h in G418‐free DMEM supplemented with 2% FBS to condition the media with WNT‐5A. Continued WNT‐5A expression was verified by Western blot analysis performed on cell lysates as well as on conditioned cell‐culture media.

Transient transfections of MDA‐MB468 cells with constitutively active Cdc42 (pRK5myc‐Cdc42L61) or dominant negative Cdc42 (pRK5myc‐Cdc42N17) were performed, using the pRK5myc empty vector as the control. When the MDA‐MB468 cells were 70–75% confluent, transfections were performed in 6‐well plates (Costar^®^, Corning Incorporated) in serum‐free DMEM (2 mL/well) supplemented with 2 μg plasmid and 6 μL Lipofectamine 2000 transfection reagent. The transfection complex medium was exchanged after 6 h with fresh DMEM supplemented with 10% FBS. After 16 h, this media was exchanged again, but with DMEM supplemented with 2% FBS, in which the cells were allowed to grow for 48 h. After this period, the cells were harvested and used for invasion or migration studies and, in parallel; the levels of Cdc42 and pERK1/2 were analyzed by Western blot.

For Cdc42 knockdown, we used siRNA. We obtained Cdc42 siRNA(h), a pool of four target‐specific 19–25 nt siRNAs from Santa Cruz Biotechnology, Inc. (sc‐29256). This pool of Cdc42 siRNA has been shown to effectively and selectively abolish Cdc42 protein in primary human fibroblast cells (Pankov et al., 2005). Silencer^®^ Select Negative control siRNA was purchased from Applied Biosystems. MDA‐MB468‐5A cells were transiently transfected with a transfection complex of 75 nM siRNA and Lipofectamine 2000 suspended in serum‐free DMEM. After 6 h, the transfection complex was removed and the medium changed to fresh DMEM containing 10% FBS. After 16 h, cells were allowed to grow in DMEM supplemented with 2% FBS for 72 h before being used for Western blotting.

### 2.3 Active Cdc42 pull‐down assay

For effective pull‐down of active Cdc42, we used a kit with human p21 activated kinase 1 (PAK1)‐Rac/Cdc (p21) binding domain (PBD) on agarose beads from Cell Biolabs (Cat. No. STA‐404). The breast cancer cells were stimulated with 0.4 μg/mL of rWNT‐5A for the stipulated time points. Prior to the rWNT‐5A treatment, cells were incubated in DMEM containing 2% FBS for 16 h. Later stimulations with rWNT‐5A were also carried out in the same medium. For the analysis of active Cdc42 in MDA‐MB468‐5A and MDA‐MB468‐EVcells, the cells were washed with PBS and grown in DMEM (containing 2% FBS) for 48 h to condition the media with WNT‐5A. The cells were washed and lyzed in the lysis buffer provided in the kit. The lysates were centrifuged and the supernatants collected and adjusted to have equal protein concentrations after estimating their protein concentrations (Bradford Reagent, Sigma). A sample from each of the adjusted supernatants was kept for equal loading detection with an α‐tubulin antibody (Santa Cruz Biotechnology Inc.) by Western blotting. Pull‐downs were performed from the remaining supernatants (approximately 1 mg of protein per sample) by addition of PAK PBD beads, thorough mixing, and incubation for 1 h at 4 °C with gentle agitation. After this incubation, the beads in each mixture were briefly centrifuged, washed, and re‐suspended in 2× Lammeli buffer, Finally, samples were boiled for 5 min under reducing conditions and loaded onto a 15% SDS‐PAGE gel. For these experiments addition of GTPγS or GDP (provided in the kit) to cell lysates were used as positive and negative controls. Following gel electrophoresis, the separated proteins were electrically transferred to PVDF membranes. Western blot analyses of these membranes were carried out as described below. Western blotting of all samples was run in parallel and analyzed for their relative content of total Cdc42 and α‐tubulin (loading controls).

#### 2.3.1 sFRP1 treatment

Recombinant Human sFRP1 was procured from R&D systems (Cat no. 5396‐SF‐025). MDA‐MB468 cells were pre‐incubated in DMEM with 2% FBS for 16 h. After this the cells incubated in the same medium for 4 h with sFRP1 (3.5 μg/ml) or rWNT‐5A (0.4 μg/ml) alone or in combination. For combinatorial treatments, cells were pretreated with sFRP1 for 1 h, before addition of rWNT‐5A. After stimulations, Cdc42 activities were analyzed as described before.

### 2.4 Cell migration

Cell culture inserts with a pore size of 8 μm were purchased from BD Labware and pre‐coated by adding 10 μg/mL Collagen I (Cat no. 354236 obtained from BD Biosciences) in PBS to the upper chamber at room temperature for 2 h, as previously described (Senger et al., 2002). At the onset of each experiment, the cells were detached with Versene and resuspended as single cells in serum‐free DMEM. For the migration experiments, we took 25,000 cells in 0.5 mL (of both cell lines) and diluted them in serum‐low DMEM (supplemented with 2% FBS). Cells were added to the upper chamber in the absence or presence of rWNT‐5A (0.4 μg/mL), and the lower chamber was filled with 0.70 mL DMEM supplemented with 10% FBS. The cells were allowed to migrate for 24 h at 37 °C in a humidified atmosphere of 5% CO_2_. The experiment was terminated by discarding the medium and fixing the cells in the filter with 4% paraformaldehyde for 10 min. Non‐invading cells on the upper side of the insert were removed by a cotton‐tipped applicator. Staining of the cells on the bottom of the membrane was performed with DAPI (300 nM in PBS) for 5 min at room temperature and washed with PBS. Membranes were excised from the inserts and mounted on slides using Dako Fluorescent Mounting Medium. We captured five or more representative fields of each membrane at 400× magnification with a fluorescent microscope (Nikon Eclipse 80i). Cells were either counted manually or with the help of the ImageJ software (National Institutes of Health, US).

### 2.5 Cell invasion

Cell invasion experiments were performed in BD Matrigel™ invasion chambers (BD Biosciences) with a membrane pore diameter of 8 μm. For the invasion experiments, we used 50,000 MDA‐MB468 cells per well or 25,000 of MDA‐MB231 cells per well, suspended in 0.5 mL serum‐low DMEM (supplemented with 2% FBS). The rest of the protocol was identical to that described in the migration assay section above.

### 2.6 Immunoblotting

Immunoblotting was performed on several different sets of samples. One set was from un‐transfected cells, treated or untreated with rWNT‐5A. Other sets were from cells stably transfected either with an empty vector (MDA‐MB468‐EV) or with WNT‐5A containing vector (MDA‐MB468‐5A). Finally, immunoblotting was performed on MDA‐MB468‐5A cells transiently transfected with either constitutively active Cdc42 (pRK5myc‐Cdc42L61) or dominant negative Cdc42 (pRK5myc‐Cdc42N17). After termination of incubation, the cells were washed with PBS and lysed in ice‐cold phosphorylation lysis buffer (PLB; 1 M Tris‐HCl pH 7.5, 0.5 M NaCl, 30 mM sodium pyrophosphate, 50 mM sodium fluoride, 0.5 M EDTA, 1.5 mM MgCl_2_, 10% Glycerol, and 1% TritonX‐100). The protein content of each sample was estimated using the Pierce^®^ BCA Protein Assay kit (Thermo). After adjustments for differences in protein content, the samples were suspended in 4× Lammeli buffer, boiled for 5 min, and loaded on SDS‐PAGE. After transfer of the separated proteins to PVDF membranes, the membranes were probed with one of these antibodies: to WNT‐5A from R&D Systems (at 1:500), to pERK and ERK (at 1:1000) from Cell Signaling Technology, to Cdc42 (at 1:1000) from BD Transductions Lab, or to α‐tubulin (at 1:10000) from Santa Cruz Biotechnology, Inc. overnight at 4 °C. After extensive washing, the membranes were incubated with either secondary goat anti‐mouse/rabbit or rabbit anti‐goat HRP‐conjugated antibodies purchased from Dako. The separated protein bands were visualized by incubating the membranes with Chemiluminescence HRP substrate (Millipore), after which the blots were imaged and analyzed in the Chemi Doc™ imaging system from Bio‐Rad.

### 2.7 Immunofluorescence

Breast cancer cells were grown on 13‐mm glass cover‐slips for 24 h at 37 °C in a humidified atmosphere of 5% CO_2._ Sixteen hours prior to stimulation with rWNT‐5A or vehicle alone, the cells were suspended in DMEM with 2% FBS; stimulations and control experiments were carried out under the same media conditions. After the stimulations, cells were washed in PBS, fixed with 4% paraformaldehyde for 20 min, and permeabilized with 0.1% TritonX‐100 for 3 min, all at room temperature. Next, the cells were washed in PBS and incubated with an anti‐pERK antibody (Cell Signaling Technology) at a 1:200 dilution. After extensive washing, a goat anti‐rabbit Alexa‐488 secondary antibody (1:400 dilution; from Molecular Probes™, Oregon, USA) was added for 45 min at 4 °C. The cells were also counter‐stained with Phalloidin‐TRITC (1:400; Sigma Aldrich) for 45 min at room temperature. Finally, the cells were rinsed with PBS and counter‐stained with DAPI (300 nM in PBS) for 2 min. For Confocal microscopy (Olympus FV10i‐O or Carl Zeiss LSM 700), the fixed and stained cells were mounted with the Dako Fluorescent Mounting Medium and viewed with a 60× objective. Quantification of the immunofluorescent staining was performed with the ImageJ software (NIH, U.S.). Briefly, the original images were converted into 8‐bit images by using the Split Channels. Plot profiles were generated using a line cross‐sectioning approach for each individual cell. The profiles generated exhibited peaks depicting membranous and cytoplasmic intensities. The area under each curve was calculated using freehand selection and measure area tools in the software. Graphs were obtained showing mean fluorescence (a.u.) corresponding to membranous and cytoplasmic localization of the protein in each cell.

### 2.8 Gelatin zymography

Gelatin zymography was done using 10% pre‐cast zymography gels according to the manufacturer's instructions (Cat no. 161‐1167, Bio‐Rad). After electrophoresis, SDS was removed by incubating the gels in a 2.5% TritonX‐100 solution and the gelatinase activities were recovered by a subsequent incubation in a Tris‐based buffer containing 10 mM calcium for 48 h. Finally, the gels were stained with Coomassie Brilliant Blue and the enzymatic activity was detected by observing the lack of gelatin protein in the sample lanes, represented by a clear band. The bands in the zymograms were inverted and integrated densitometric analyses were carried out with a Chemi Doc™ imaging system from Bio‐Rad and the ChemiImager 4400 software (Alpha Innotech).

### 2.9 Statistical analysis

Data are expressed as the mean ± standard error of mean. Each experiment was repeated at least 3 times, as indicated in the figure legends. Statistical evaluations were performed either by a two‐tailed Student's *t*‐test (*p* values <0.05 were considered significant), or by ANOVA together with Dunnett's Multiple Comparison test (*p* < 0.01 was considered significant). All the statistical significances and plots were generated using the GraphPad Prism program, version 5.00 for Windows.

## 3 Results

### 3.1 WNT‐5A activates Cdc42 in breast cancer cells

**Figure 1:**
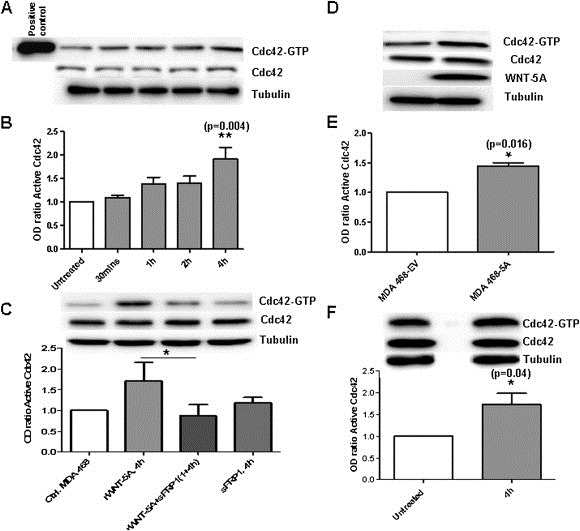
WNT‐5A activates Cdc42 in breast cancer cells. (A) MDA‐MB468 cells were stimulated with rWNT‐5A (0.4 μg/mL) for the indicated periods of time (0–4 h) and lysed. The lysate was either directly analyzed by Western blot for its content of total Cdc42 or α‐tubulin or used for GST‐PAK1 PBD pull down and subsequent analysis of Cdc42 (Cdc42‐GTP). (B) Quantifications of Cdc42‐GTP in rWNT‐5A stimulated MDA‐MB468 cells were carried out by calculating Integrated Density Values and normalizing it against total Cdc42 levels. (C) In order to confirm the specificity of the WNT‐5A‐induced Cdc42 activation, analysis of active Cdc42 (Cdc42‐GTP) was performed after stimulating the parental MDA‐MB468 cells with rWNT‐5A (0.4 μg/mL) in the absence of presence of sFRP1 (3.5 μg/mL). (D) Lysates of stably transfected MDA‐MB468‐5A were either directly analyzed by Western blot for content of total Cdc42 or α‐tubulin or used for GST‐PAK1 PBD pull down and subsequent analysis of Cdc42 (Cdc42‐GTP) and (E) Quantifications of Cdc42‐GTP in MDA‐MB468‐5A cells were carried out by calculating Integrated Density Values. (F) MDA‐MB231 cells were stimulated with rWNT‐5A (0.4 μg/mL) for 4 h and analyzed as in panels (A–F). Statistical comparisons between means were made with one‐way ANOVA (with Dunnett's Multiple Comparison test for post analysis; B), and Student's t‐test (C, E and F). All error bars represent standard error of the mean (n = 4). *p < 0.05; **p < 0.01.

We have previously demonstrated that rWNT‐5A activated Cdc42 in non‐cancerous ductal breast epithelial HB2 cells, where the WNT‐5A protein level was low because the cells were WNT‐5A antisense‐transfected (Dejmek et al., 2006). Moreover, we also reported that in resting HB2 cells expressing WNT‐5A, Cdc42 activities were higher than in cells not expressing WNT‐5A (Dejmek et al., 2006). In the current set of experiments, we stimulated MDA‐MB468 breast cancer cells with rWNT‐5A at a dose of 0.4 μg/mL at various times (Figure 1A). Glutathione S‐transferase (GST) PAK1 PBD beads were used to pull down GTP‐bound Cdc42 (the active form). rWNT‐5A significantly activated Cdc42 in MDA‐MB468 cells in a time‐dependent manner (Figure 1A). The results were verified by analyzing integrated densitometric values of active Cdc42 (Figure 1B). In order to relate the rWNT‐5A induced Cdc42 response quantitatively with maximal‐receptor mediated response in these cells, we stimulated MDA‐MB468 cells with 100 ng/ml of EGF (epidermal growth factor) for 1, 5, 15 and 30 min. We found that EGF induced a 3‐fold increase in Cdc42‐GTP within 1 min (as shown in Supplementary Figure 1). To confirm the specificity of the rWNT‐5A induced Cdc42 activation, we utilized a WNT‐5A antagonist i.e. recombinant sFRP1. We observed that sFRP1 significantly neutralized the increased activity of Cdc42 that was mediated by rWNT‐5A in MDA‐MB468 cells (Figure 1C). To further confirm that WNT‐5A induces an increase in Cdc42 activity, we generated MDA‐MB468 cells stably transfected with pcDNA3.1‐WNT‐5A plasmid. We detected significant increased levels of WNT‐5A mRNA and protein in MDA‐MB468 cells transfected with the pcDNA3.1‐WNT‐5A plasmid compared to empty pcDNA3.1 transfected cells (Supplementary Figure 2A). The release of WNT‐5A from these cells was confirmed by Western blot analysis of the media (Supplementary Figure 2B). We observed significantly higher levels of active Cdc42 in MDA‐MB468 cells that were stably expressing the WNT‐5A protein (MDA‐MB468‐5A) than in the empty‐vector transfected cells (MDA‐MB468‐EV; Figure 1D and E). To rule out the possibility that the increase in active Cdc42 induced by WNT‐5A is a unique property of MDA‐MB468 breast cancer cells, we employed MDA‐MB231 in our experiments. rWNT‐5A (0.4 μg/mL at 4 h) significantly activated Cdc42 also in MDA‐MB231 cells (Figure 1F). In MDA‐MB231 cells transfected with the pcDNA3.1‐WNT‐5A plasmid, we detected an increased WNT‐5A transcript level in transfected MDA‐MB231 cells, but the lysates of these transfected cells showed little or no expression of the WNT‐5A protein (Supplementary Figure 2C). Consequently, we could not study the effect of WNT‐5A transfection on Cdc42 activity in this cell line.

### 3.2 WNT‐5A inhibits breast cancer cell migration and invasion

**Figure 2:**
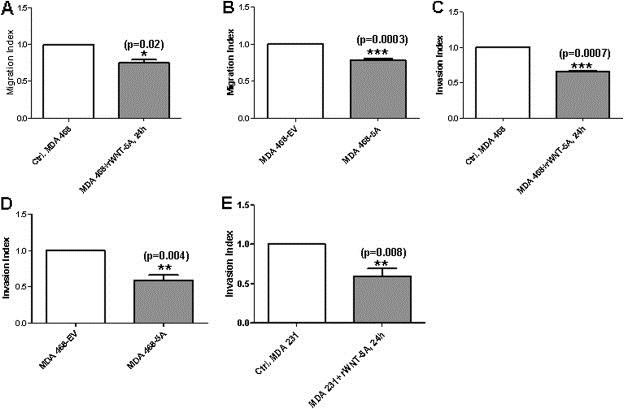
WNT‐5A significantly decreases the migration and invasion of breast cancer cells. The Trans‐well migration assay was performed using inserts coated with Collagen‐I and the invasion assay was carried out using BD Matrigel™ invasion chambers (as described in Materials and methods). (A) MDA‐MB468 cells were either untreated or treated with rWNT‐5A (0.4 μg/mL) in the upper chamber of the Trans‐well and allowed to migrate for 24 h. Cells that had migrated to the bottom of the membrane were counted manually after staining with DAPI. The migration of rWNT‐5A exposed cells was normalized against migration of vehicle exposed control cells. (B) MDA‐MB468‐EV and MDA‐MB468‐5A cells were placed in the upper chamber of the Trans‐well and allowed to migrate for 24 h. Cells that had migrated to the bottom of the membrane were counted manually after staining with DAPI. The migration of MDA‐MB468‐5A cells was normalized against migration of MDA‐MB468‐EV control cells. (C) MDA‐MB468 cells were either untreated or treated with rWNT‐5A (0.4 μg/mL) in the upper Matrigel invasion chamber and allowed to migrate for 24 h. Cells that had invaded to the bottom of the membrane were counted manually after staining with DAPI. The invasion of rWNT‐5A exposed cells was normalized against invasion of vehicle‐exposed control cells. (D) MDA‐MB468‐EV and MDA‐MB468‐5A cells were placed in the upper Matrigel invasion chamber and allowed to migrate for 24 h. Cells that had invaded to the bottom of the membrane were counted manually after staining with DAPI. The invasion of MDA‐MB468‐5A cells was normalized against invasion of MDA‐MB468‐EV control cells. (E) MDA‐MB231 cells were treated or not with rWNT‐5A (0.4 μg/mL) in the upper Matrigel invasion chamber and allowed to migrate for 24 h. Cells that had invaded to the bottom of the membrane were counted manually after staining with DAPI. The invasion of rWNT‐5A‐exposed cells was normalized against invasion of vehicle‐exposed control cells. The control used for all rWNT‐5A stimulations was PBS supplemented with 0.1% BSA. Statistical comparisons between means were made with Student's t‐test (A–E). All error bars represent standard error of the mean (n = 4). *p < 0.05; **p < 0.01; ***p < 0.001.

It is well established that Cdc42, a member of the RhoGTPase family, positively regulates cancer cell migration (Heasman and Ridley, 2008; Ridley et al., 1999; Whale et al., 2011). Because our results indicated that WNT‐5A comprehensively activates Cdc42 in breast cancer cells, we next performed Trans‐well migration and invasion assays with breast cancer cells in the absence or presence of WNT‐5A. For the Trans‐well migration assay, we employed a thin film of collagen by adding a collagen‐I solution (10 μg/mL) in the inner well for 2 h. MDA‐MB468 cells exposed to rWNT‐5A migrated less than the vehicle‐treated control (Figure 2A). The same phenomenon was observed in MDA‐MB468‐5A, as compared to control MDA‐MB468‐EV cells (Figure 2B). We found similar effects of WNT‐5A on MDA‐MB468 cells invasion, as the penetration of the cells through the matrigel was significantly inhibited in cells treated with rWNT‐5A(0.4 μg/mL for 24 h) when compared to vehicle‐treated control cells (Figure 2C) and in MDA‐MB468‐5A cells in comparison with MDA‐MB468‐EV control cells (Figure 2D). We also observed that the invasion of MDA‐MB231 cells was significantly reduced in the presence of rWNT‐5A (0.4 μg/mL) for 24 h when compared to vehicle‐treated (0.1% BSA in PBS) controls (Figure 2E). These results indicate that WNT‐5A restricts the migration and invasion of MDA‐MB468 and MDA‐MB231 cells.

### 3.3 WNT‐5A modulates the activation of pERK1/2 in breast cancer cells

**Figure 3:**
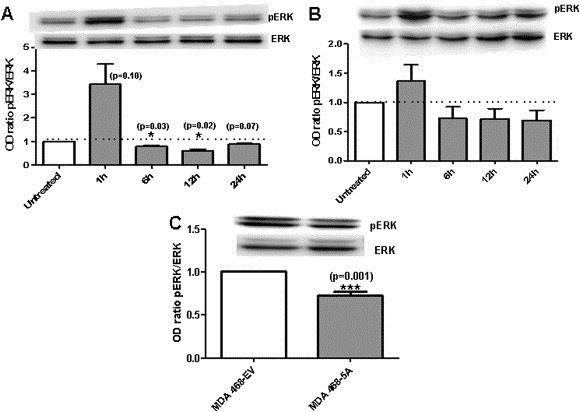
WNT‐5A modulation of ERK1/2 activity. (A) ERK1/2 activation was analyzed in MDA‐MB468 cells either not stimulated or stimulated with rWNT‐5A (0.4 μg/mL) for 1 h, 6 h, 12 h, or 24 h, after which the cells were lysed in PLB buffer (as described in Materials and methods). Western blot analyses were performed with a pERK1/2 and a total ERK1/2 antibody. Quantifications of pERK1/2 in non‐stimulated and rWNT‐5A‐stimulated MDA‐MB468 cells were carried out by calculating Integrated Density Values and normalizing it against total ERK levels. (B) ERK1/2 activation was analyzed in MDA‐MB231 cells either not stimulated or stimulated with rWNT‐5A (0.4 μg/mL) for 1 h, 6 h, 12 h, or 24 h, after which cells were lysed and analyzed, followed by quantification as in panel A. (C) ERK1/2 activation was analyzed in MDA‐MB468‐EV cells and MDA‐MB468‐5A cells as in panel A. Quantifications of pERK1/2 in MDA‐MB468‐EV and MDA‐MB468‐5A cells were carried out by calculating Integrated Density Values and normalizing it against total ERK levels. Statistical comparisons between means were made with Student's t‐test (A, B, and C). All error bars represent standard error of the mean (n = 4). *p < 0.05; ***p < 0.001.

The ERK pathway is one of the most intensively studied mammalian MAPK pathways. It is deregulated in approximately one‐third of all human cancer cases (Reddy et al., 2003), which attests to its importance. There were two reasons for studying ERK1/2 signaling in the current context. First, it drives breast cancer cell migration and invasion (Chen et al., 2009; von Thun et al., 2012) and second, it can be regulated by Cdc42 (Deroanne et al., 2005; Zuo et al., 2011). Breast cancer cells (MDA‐MB468 and MDA‐MB231) were stimulated with rWNT‐5A (0.4 μg/mL) in a time‐dependent manner. The phosphorylation of ERK1/2 were analyzed after 1 h, 6 h, 12 h, and 24 h by Western blotting using a polyclonal antibody specific to phosphorylated sites at Thr202/Tyr204. Phosphorylation of these sites is coupled to the activity of ERK1/2 and thus a reliable and indirect method for estimating ERK1/2 activity (Osabe and Negishi, 2011; Procaccia et al., 2010). Based on this, we have from here onwards referred to our determinations of phosphor‐ERK1/2 levels as ERK1/2 activities.Both breast cancer cell lines had the same activation kinetics of ERK1/2 when stimulated with rWNT‐5A. WNT‐5A initially induced activation of ERK1/2 followed by a phase in which the activity fell below the basal level (Figure 3A and B; Supplementary Figure 3A). These results are validated by statistical analysis of pERK/ERK integrated‐density values of MDA‐MB468 cells treated with or without rWNT‐5A. The results demonstrated statistical decreases after 6 h and 12 h of rWNT‐5A stimulation (Figure 3A). Complementary experiments reveals that this pERK1/2 activation in MDA‐MB468 cells occurred already after 5 m (Supplementary Figure 3B). We conclude that the initial increase was Ca^2+^ dependent, because when BAPTA (1,2‐bis(o‐aminophenoxy)ethane‐ N,N,N′,N′‐tetraacetic acid)‐loaded MDA‐MB468 cells were stimulated with rWNT‐5A, the initial increase in ERK1/2 activity was diminished (Supplementary Figure 3C).

To further verify the rWNT‐5A mediated decrease in ERK1/2, we analyzed ERK1/2 activity in MDA‐MB468 cells transfected and expressing the WNT‐5A protein (MDA‐MB468‐5A). We observed a statistically significant decrease in ERK1/2 activity in MDA‐MB468‐5A cells compared with control MDA‐MB468‐EV cells by Western blot (Figure 3C).

**Figure 4:**
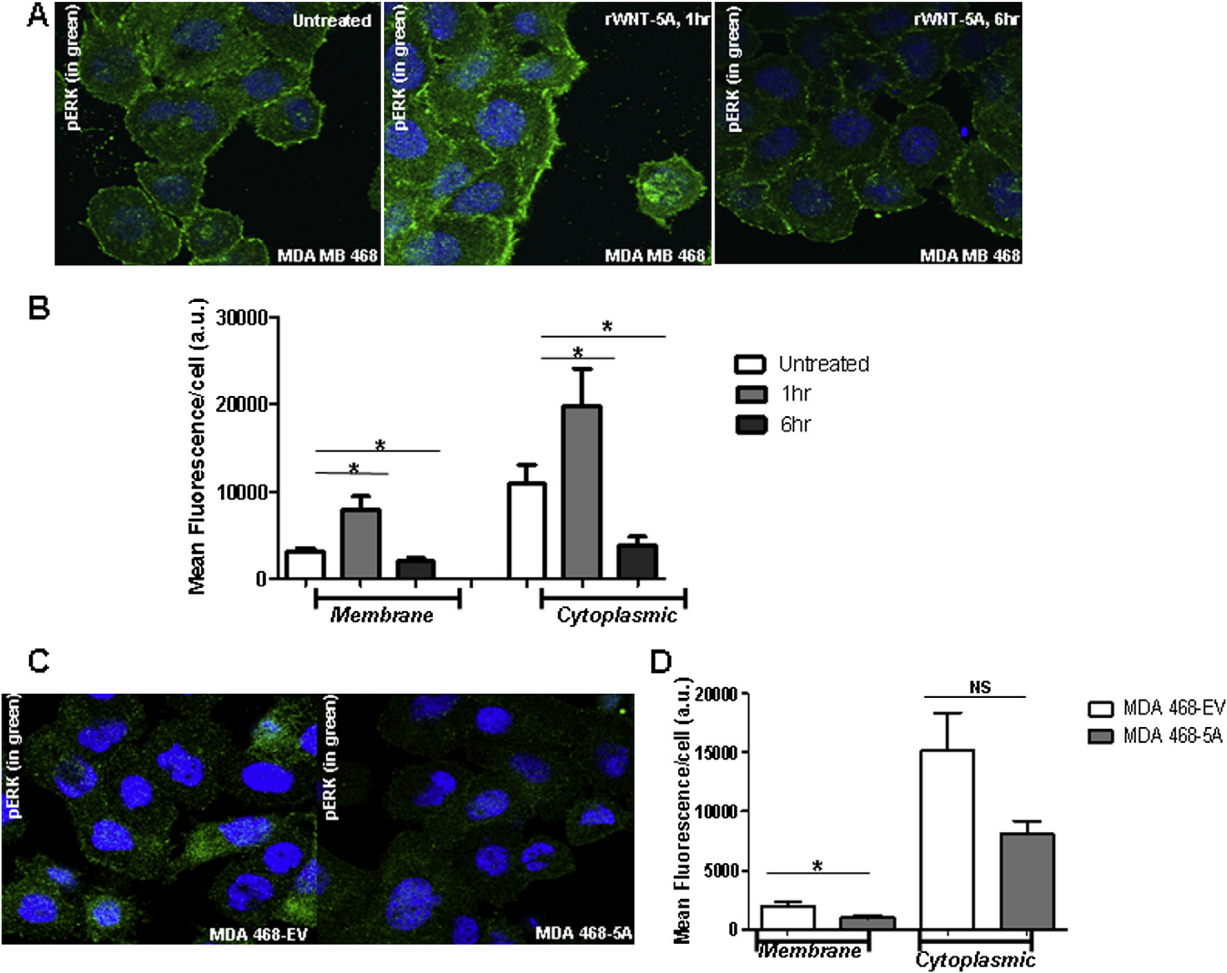
Immunofluorescence and Semi‐quantification of pERK1/2 staining. (A) Immunofluorescence staining of pERK1/2 in MDA‐MB468 cells not treated with rWNT‐5A or treated for 1 h or 6 h. For visualization, a secondary goat anti‐rabbit Alexa‐488 labeled antibody was used together with DAPI counterstaining. (B) Semi‐quantification of these pERK1/2 fluorescent stainings were performed as described in Materials and methods. For each experiment between 10 and 15 cells were evaluated per slide. Each experiment was performed in duplicate. (C) Immunofluorescence staining of pERK1/2 in MDA‐MB468‐EV and MDA‐MB468‐5A cells was visualized in same way as described in panel A. (D) Semi‐quantification of these pERK1/2 fluorescent stainings were performed in the same way as in panel B. Statistical comparisons between means were made with Student's t‐test (B and D). All error bars represent standard error of the mean (n = 3). *p < 0.05.

We also used confocal microscopy of MDA‐MB468 cells to confirm the WNT‐5A changes in ERK1/2 activation. rWNT‐5A induced a marked increase in pERK1/2 staining within 1 h. Semi‐quantitative estimation of the fluorescent intensity revealed a significant increase in pERK1/2 staining at the membrane as well as in the cytoplasm, as compared to untreated cells. However, at 6 h we observed a significant decrease in the staining intensity of pERK1/2 both at the membrane and in the cytoplasm (Figure 4A and B). So, these data from confocal microscopy confirm the results from the Western blotting (Figure 3). Also, the MDA‐MB468 cells transfected and expressing the WNT‐5A protein (MDA‐MB468‐5A) showed a marked reduction in pERK1/2 staining, supporting the Western blot findings (Figure 4C and D).

These data suggest that prolonged exposure of cells to WNT‐5A also impaired ERK1/2 activity. The experiments with the MEK1/2 inhibitor (U0126) showed that inadequate ERK1/2 signaling impaired migration of MDA‐MB468 cells (Supplementary Figure 3D), suggesting its direct role in migration of MDA‐MB468 cells. Together these data indicate that apart from an initial increase, WNT‐5A confine ERK1/2 activity in breast cancer cells and that impaired ERK1/2 activity renders breast cancer cells less migratory and invasive.

### 3.4 Silencing of Cdc42 upregulates ERK1/2 activity in MDA‐MB468‐5A cells

**Figure 5:**
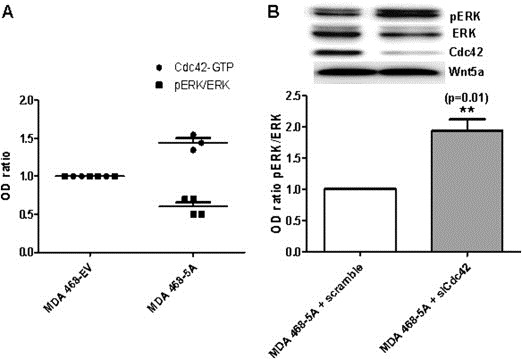
Inverse association between Cdc42 and ERK1/2 activities in MDA‐MB468‐5A cells. (A) Graphical representation of Integrated Density Values of Cdc42‐GTP and pERK1/2 levels analyzed by Western blotting in MDA‐MB468‐5A and MDA‐MB468‐EV cells. (B) MDA‐MB468‐5A cells were treated with either 75 nM of Cdc42 siRNA cocktail (as described in Materials and methods) or the same dose of scrambled siRNA. After this treatment, the cells were lysed in PLB buffer and analyzed for their content of Cdc42, pERK1/2, ERK1/2, and WNT‐5A proteins by Western blotting. Quantifications of pERK1/2 in MDA‐MB468‐5A cells treated with Cdc42 siRNA or scrambled siRNA were carried out by calculating Integrated Density Values of the pERK1/2 bands and normalizing it against that of total ERK. Statistical comparisons between means were made with Student's t‐test. All error bars represent standard error of the mean (n = 6). **p ≤ 0.01.

MDA‐MB468 cells transfected to express the WNT‐5A protein had elevated levels of the active GTP‐bound form of Cdc42 and reduced levels of active ERK1/2 (Figure 5A). These changes occurred simultaneously with a reduction of basal cell migraton and invasion in MDA‐MB468‐5A cells compared to the control MDA‐MB468‐EV cells. These results led us to ask whether WNT‐5A‐induced suppression of ERK1/2 activity is mediated via an increased Cdc42 activity in these cells. To answer this question, we employed a siRNA knockdown approach. The transfection method was optimized on MDA‐MB468‐5A cells (*data not shown*) after which the cells were transfected with 75 nM siRNA specific for Cdc42 mRNA. MDA‐MB468‐5A cells transfected with siRNA against Cdc42 mRNA had significant increased activity of ERK1/2, as compared to control MDA‐MB468‐5A cells treated with scramble siRNA (Figure 5B). The results suggest that in MDA‐MB468‐5A cells, the WNT‐5A‐induced inhibition of ERK1/2 activity is mediated via Cdc42.

### 3.5 Transfection of Cdc42 mutant forms interfere with migration and invasion of MDA‐MB468 cells

**Figure 6:**
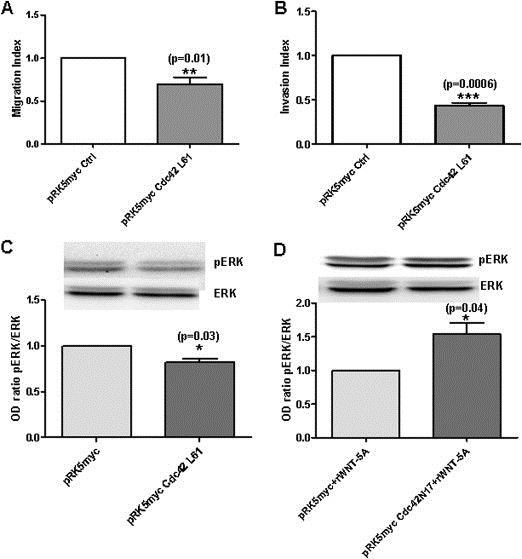
Effects of Cdc42 mutants on basal cell migration, invasion and ERK1/2 activity in MDA‐MB468 cells. (A) MDA‐MB468 cells transfected with either dominant active Cdc42 (pRK5myc‐Cdc42L61 plasmid) or empty vector (pRK5myc‐EV plasmid) were placed in the upper chamber of the Trans‐well and allowed to migrate for 24 h. Cells that had migrated to the bottom of the membrane were counted manually after staining with DAPI. The migration of MDA‐MB468 cells transfected with pRK5myc‐Cdc42L61 plasmid was normalized against migration of pRK5myc‐EV transfected control cells. (B) MDA‐MB468 cells transfected with pRK5myc‐Cdc42L61 or control pRK5myc‐EV were placed in the upper Matrigel invasion chamber and allowed to migrate for 24 h. Cells that had invaded to the bottom of the membrane were counted manually after staining with DAPI. The invasion of MDA‐MB468 cells (transfected with pRK5myc‐Cdc42L61) was normalized against invasion of pRK5myc‐EV transfected control cells. (C) Quantification of pERK1/2 activity in MDA‐MB468 cells transfected with pRK5myc‐Cdc42L61 or control pRK5myc‐EV was carried out by calculating Integrated Density Values of the pERK1/2 band and normalizing it against that of total ERK. (D) ERK1/2 activity was analyzed in MDA‐MB468 cells transfected with either dominant negative Cdc42 (pRK5myc‐Cdc42N17) or pRK5myc‐EV cells followed by rWNT‐5A stimulations for 24 h after which the cells were lysed and analyzed by Western blotting. Quantification of pERK1/2 in MDA‐MB468 cells transfected with either pRK5myc‐EV (control) or pRK5myc‐Cdc42N17 was carried out by calculating Integrated Density Values of the pERK1/2 band and normalizing it against that of total ERK. Statistical comparisons between means were made with Student's t‐test. All error bars represent standard error of the mean (n = 3). *p < 0.05; **p ≤ 0.01; ***p < 0.001.

To further confirm that Cdc42 activity inhibits migration and invasion of MDA‐MB468 cells, we took a complementary approach in which we transiently transfected parental MDA‐MB468 cells with either dominant positive or dominant negative Cdc42 mutants (Cdc42L61 and Cdc42N17) and assessed changes in migration, invasion and ERK1/2 activity. The expression of Cdc42 mutants were confirmed by immunoblotting and enabled a comparison with basal Cdc42 levels (Supplementary Figure 4A and B). MDA‐MB468 cells transfected with the constitutively active Cdc42 mutant (Cdc42L61) exhibited significantly less migration and invasion than the control cells (Figure 6A and B). In addition, the inhibition of migration and invasion of Cdc42L61 transfected MDA‐MB468 cells occurred in parallel with a reduced ERK1/2 activity (Figure 6C). In another approach, we transiently transfected MDA‐MB468 cells with the dominant negative Cdc42 mutant (Cdc42N17) (Edlund et al., 2002) and studied their response to a 24 h incubation with rWNT‐5A. In comparison with control cells, cells transfected with Cdc42N17 had increased activity of ERK1/2 in response to rWNT‐5A stimulation (Figure 6D). These results support our conclusion that increased Cdc42 activity reduces the activity of ERK1/2 as well as breast cancer cell migration and invasion.

### 3.6 WNT‐5A inhibits the invasive migration of MDA‐MB468 cells by inhibiting Gelatinase‐B (MMP9)

**Figure 7:**
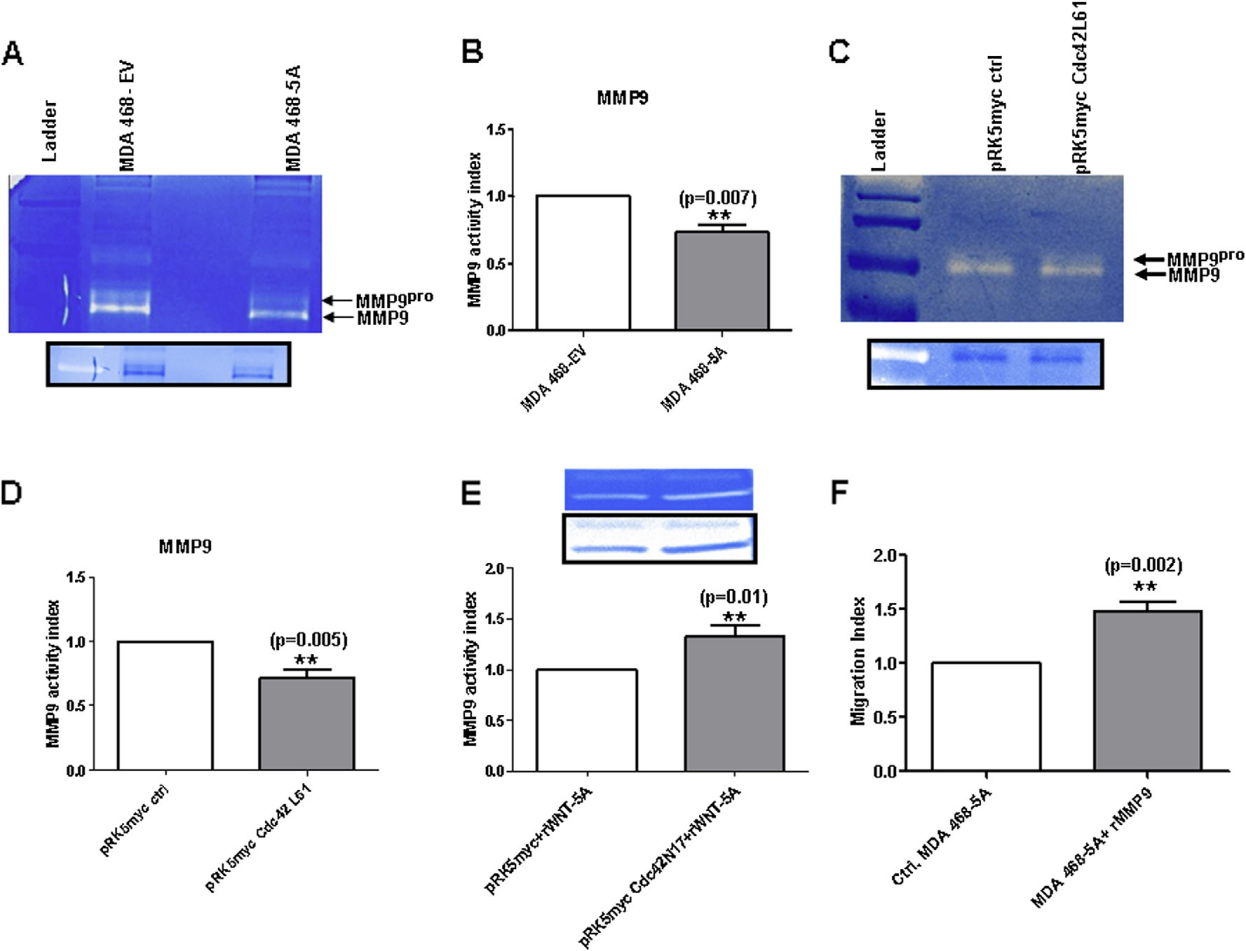
WNT‐5A and Cdc42 impairs MMP9 activity. (A) Gelatin‐zymography was performed on media from either MDA‐MB468‐5A or MDA‐MB468‐EV cells as described in Materials and methods. MMP9 activity was visualized as transparent bands on Coomassie blue‐stained gels. The gel images were inverted (as shown in the inserts) and quantitated by determining the integrated densitometric values. (B) Quantification of MMP9 activity in media from MDA‐MB468‐5A cells was performed by calculating the integrated densitometric value of the inverted band and normalizing it against that obtained from MDA‐MD468‐EV cells. (C) Gelatin‐zymography was performed on media from MDA‐MB468 cells transfected with either pRK5myc‐Cdc42L61 or pRK5myc‐EV (control cells). MMP9 activity was visualized and quantitated as in panel A. (D) Quantification of MMP9 activity in media from MDA‐MB468 cells transfected with pRK5myc‐Cdc42L61 was performed by calculating the integrated densitometric value of its inverted band and normalizing it against that obtained from pRK5myc‐EV transfected MDA‐MB468 cells. (E) Quantification of MMP9 activity in the media from MDA‐MB468 cells transiently transfected with pRK5myc‐Cdc42N17 (dominant negative mutant of Cdc42) followed by rWNT‐5A stimulation for 24 h. rWNT‐5A stimulated MDA‐MB468 cells (transfected with pRK5myc vector) served as control. (F) MDA‐MB468‐5A cells were either untreated or treated with active human rMMP9 (from Calbiochem, 20 ng/mL) by its addition to the upper chamber of the Trans‐well after which the cells were allowed to migrate for 24 h. Cells that had migrated to the bottom of the membrane were counted manually after staining with DAPI. The migration of rMMP9 exposed cells was normalized against the migration of vehicle‐exposed control cells. Statistical comparisons between means were made with Student's t‐test. All error bars represent standard error of the mean (n = 7). **p < 0.01.

Our next goal was to locate possible downstream targets of Cdc42‐ERK1/2 signaling that could be linked to breast cancer cell migration and invasion. A possible candidate for this linkage is gelatinase‐B (MMP9), as it is activated by ERK1/2 (Beliveau et al., 2010; Moshal et al., 2006) and can modulate cell migration (Chen et al., 2009; Rolli et al., 2003). We first investigated the activity of MMP9 in MDA‐MB468‐5A cells through Gelatin‐Zymography. The activity of MMP9 was significantly reduced in the media from MDA‐MB468‐5A cells compared to the media from MDA‐MB468‐EV control cells (Figure 7A). The quantitative analysis confirmed that the activity of MMP9 was significantly reduced in WNT‐5A‐expressing MDA‐MB468 cells (Figure 7B). In order to further verify these data, MMP9 protein expression was analyzed in MDA‐MB468‐5A and MDA‐MB468‐EV cells by Immunofluorescence Microscopy. We observed an overall decrease in MMP9 protein expression in MDA‐MB468‐5A cells, as compared to control cells (Supplementary Figure 5). The decrease in both expression level and activity of MMP9 in MDA MB468‐5A cells compared to control cells suggests that this gelatinase is involved in WNT‐5A‐modulated breast cancer cell migration and invasion. We analyzed the media collected from parental MDA‐MB468 cells transiently transfected with the constitutively active mutant of Cdc42 (Cdc42L61) for MMP9 activity to determine if Cdc42 signaling suppressed gelatinase‐B activity. The activity of MMP9 in Cdc42L61‐transfected MDA‐MB468 cells was significantly less than in control cells (Figure 7C and D). With an alternative approach, we demonstrated a statistically significant rWNT‐5A‐induced increase in MMP9 activity in media from parental MDA‐MB468 cells transiently transfected with the dominant negative Cdc42 mutant (Cdc42N17; Figure 7E). Further evidence that MMP9 is an important downstream mediator of WNT‐5A‐regulated breast cancer cells migration comes from experiments in which we utilized active human rMMP9. rMMP9 (20 ng/mL) was added to MDA‐MB468‐5A cells to counteract the demonstrated ability of WNT‐5A to impair MMP9 activity (Figure 7B). In accordance with our previous results, addition of rMMP9 increased the migration of MDA‐MB468‐5A cells in comparison to vehicle‐treated control cells (Figure 7F).

## 4 Discussion

In the present study, WNT‐5A activated Cdc42 in MDA‐MB468 and MDA‐MB231 breast cancer cell lines (both ATCC‐certified and ‐characterized). Treatment with rWNT‐5A for 4 h, indicate that Cdc42 is one of the signaling proteins activated by WNT‐5A in these cells. This increase in Cdc42 activity persisted in this study, as shown by elevated Cdc42 activity in resting MDA‐MB468‐5A cells that are continuously expressing and releasing the WNT‐5A protein. The specificity of the WNT‐5A‐induced Cdc42 activation was confirmed by using recombinant human sFRP1, which significantly inhibited the rWNT‐5A induced Cdc42 activation when given in a 1:10 ratio. In the next set of experiments, we performed Trans‐well migration and invasion assays to test the effect of increased activity of Cdc42 in breast cancer cells as Cdc42 is known to positively regulate cell proliferation, migration, and invasion of tumor cells (Melendez et al., 2010; Stengel and Zheng, 2011). Despite this, a WNT‐5A‐mediated increase in Cdc42 occurred in parallel with decreased migration and invasion of MDA‐MB468 cells, regardless of whether the cells had been treated with rWNT‐5A or transfected to express WNT‐5A (MDA‐MB468‐5A). Furthermore, rWNT‐5A‐treated MDA‐MB231 cells had significantly reduced invasion capability. Transient transfection of constitutively active Cdc42 mutant (Cdc42L61) alone inhibited the basal migration and invasion of MDA‐MB468 cells. There is a recent report of an anti‐migratory function of Cdc42 in aggressive breast cancer cells (Zuo et al., 2011), a result that supports our finding. In this report, the authors used a siRNA approach to demonstrate that knocking down of Cdc42 led to an increase in the basal migration and invasion of MDA‐MB231 and C3L5 cells. Consequently, we highlighted Cdc42 as a downstream mediator of non‐canonical WNT‐5A signaling in breast cancer cells and suggest an essential role of Cdc42 activation in reducing breast cancer cell migration and invasion.

Cdc42 has been shown to control the activation of ERK1/2 in a cell‐type specific manner (Deroanne et al., 2005; Szczur et al., 2006; Zugasti et al., 2001; Zuo et al., 2011), so we explored the possibility that WNT‐5A regulates ERK1/2 activity through Cdc42. It has also been shown that MAPKs play an important role in cell migration (Huang et al., 2004) and, in particular, ERK1/2 is an integral signal in breast cancer cell migration (Chen et al., 2009). In our current experiments, we stimulated both MDA‐MB468 and MDA‐MB231 cells with rWNT‐5A and followed the activation kinetics of ERK1/2 for 24 h, using a phospho‐specific pERK1/2 antibody to monitor the activity of this kinase (Osabe and Negishi, 2011). We observed identical ERK1/2 activation kinetics in both cell lines: upon rWNT‐5A stimulation there was an initial and transient increase in the activity of ERK1/2, peaking at 1 h, and after that, the activity declined and remained below the basal level for at least 24 h. The initial transient increase was Ca^2+^ dependent as the WNT‐5A‐induced increase in ERK1/2 activity was neutralized when the cells were loaded with BAPTA, a Ca^2+^ chelator. These results agree with previous findings in other cell types that Ca^2+^ positively regulates activation of ERK1/2 (Chao et al., 1992; Estrada et al., 2003; Moshal et al., 2006). rWNT‐5A triggers a Ca^2+^‐dependent ERK activation with rapid onset and transient duration, prior to WNT‐5A‐induced statistically significant increase in Cdc42 activity. The significant decrease in ERK1/2 activity after 6 h and 12 h in rWNT‐5A‐stimulated MDA‐MB468 cells fits well with the decrease in ERK1/2 activity level in MDA‐MB468‐5A cells. The immunofluorescence and semi‐quantitative data confirmed our findings from the Western blot experiments. Together, these data point to the ability of WNT‐5A signaling to restrict the activity of ERK1/2 via a Cdc42‐dependent mechanism that results in impaired migration and invasion of breast cancer cells. This proposal is in agreement with the observed increase in ERK1/2 activity and cell migration in Cdc42‐depleted MDA‐MB231 cells (Zuo et al., 2011). However, a recent study demonstrated increased migration of breast cancer cells in response to rWNT‐5A treatment after a very short stimulation period (Zhu et al., 2012). It is possible that short‐term stimulation with rWNT‐5A can elicit an early and transient increase in cell migration as suggested by our finding of an early and transient increase in ERK1/2 activity. However in a breast cancer context the effects of prolonged WNT‐5A exposure on cell migration would be more relevant for how WNT‐5A affects breast cancer metastasis. In the present study, we tried to mimic the *in vivo* situation of prolonged WNT‐5A exposure by transfecting MDA‐MB468 cells with a WNT‐5A plasmid. Interestingly enough, these transfected cells exhibit increased Cdc42 activity, decreased ERK1/2 activity, and decreased migration and invasion.

The effects of WNT‐5A on Cdc42 and ERK1/2 suggest an inverse relationship between Cdc42 and ERK1/2 in MDA‐MB468 cells, a suggestion that is further supported by the finding that inhibition of Cdc42 resulted in increased ERK1/2 activity (Zugasti et al., 2001; Zuo et al., 2011). In MDA‐MB468‐5A cells, which have high endogenous Cdc42 activity, silencing of Cdc42 induced a significant increase in ERK1/2 activity, suggesting that WNT‐5A‐mediated Cdc42 signaling suppresses the activity of ERK1/2 in these cells. As previously discussed, transient transfection of MDA‐MB468 cells with the constitutively active Cdc42 mutant (Cdc42L61) impaired their migration and invasion and was accompanied by a significant decrease in ERK1/2 activity. In a reverse approach, we transfected these cells with a dominant negative mutant of Cdc42 (Cdc42N17) and noted that rWNT‐5A stimulation caused increased ERK1/2 activity. On the basis of these results, we conclude that WNT‐5A impairs breast cancer cell migration and invasion by increasing the activity of Cdc42, thereby decreasing ERK1/2 activity.

MMP9 activity has been implicated in breast cancer cell migration (Rolli et al., 2003) and ERK1/2 activity has been positively associated with increased MMP activity (Yao et al., 2004). This information led us to next investigate the role of MMP9 in WNT‐5A‐impaired breast cancer cell migration and invasion. Cell‐culture medium collected from MDA‐MB468‐5A cells revealed a significant reduction in MMP9 activity compared to empty‐vector transfected control cells. Similarly, immunofluorescence staining clearly revealed downregulation of MMP9 expression in MDA‐MB468‐5A cells compared to empty vector control cells and there was decreased MMP9 activity in the culture medium from parental MDA‐MB468 cells transfected with constitutive active Cdc42 mutant (Cdc42L61). In an alternate approach, we observed a WNT‐5A‐triggered increase in MMP9 activity when parental MDA‐MB468 cells were transiently transfected with dominant negative Cdc42 mutant (Cdc42N17). These data indicate that WNT‐5A impairs MMP9 activity via Cdc42 signaling. Finally, to show that the WNT‐5A‐induced inhibition of MDA‐MB468 breast cancer cell migration is dependent on reduced MMP9 activity, we studied MDA‐MB468‐5A cell migration in the absence and presence of active human recombinant MMP9. The addition of rMMP9 to MDA‐MB468‐5A cells led to increased migration compared to vehicle‐treated control cells. Taken together, these results lead us to conclude that WNT‐5A‐mediated impaired breast cancer cell migration and invasion is at least in part explained by a reduced release of MMP9. Interestingly, it has recently been demonstrated that WNT‐5A induced MMP9 mRNA expression through a ERK1/2 signaling mechanism in microglia (Halleskog et al., 2012), which appears contrary to our present findings on breast cancer cells. If the WNT‐5A‐induced MMP9 mRNA expression relates to an increase in MMP9 activity this is another good example of how WNT‐5A can have different and opposing effect depending on the cellular context.

In human breast cancer tissue it has been shown that WNT‐5A protein expression accompanied prolonged disease‐free survival and overall survival. In agreement, we here provide evidence that although WNT‐5A activates Cdc42, it causes a downstream decrease in ERK1/2 and MMP9 activities those results in impaired breast cancer cell migration and invasion. We believe that WNT‐5A‐modulated Cdc42‐ERK1/2 signaling plays an integral part in restricting breast cancer progression and metastasis. Future studies are warranted to identify potential additional downstream targets complementing the observed effect on MMP9 activity.

## Competing interests

L.A. and T.A. are shareholders of WNT‐Research and T.A. is part‐time Chief Scientific Officer of WNT‐Research. This does not alter the authors' adherence to all the policies on sharing data and materials as stated for the *Molecular Oncology*.

## Supporting information



The following are the supplementary data related to this article:

Figure S1 EGF‐mediated activation of Cdc42 in MDA‐MB468. MDA‐MB468 cells were stimulated with EGF(100 ng/ml) for the indicated periods of time (0–30 m). Following these stimulations the cells were lysed and either directly analyzed by Western blotting for its content of total Cdc42 or α‐tubulin or used for GST‐PAK1 PBD pull down and subsequent analysed for the level of active Cdc42 (Cdc42‐GTP). Quantifications of Cdc42‐GTP in EGF stimulated MDA‐MB468 cells were carried out by calculating the Integrated Density Values and normalizing them against total Cdc42 levels. The error bars represent standard error of the mean (n = 3).

Figure S2 Establishment of breast cancer cells stably expressing the WNT‐5A protein. (A) A representative Western blot showing WNT‐5A protein expression in MDA‐MB468 cells stably transfected with the pcDNA3.1(+)‐WNT‐5A plasmid (MDA‐MB468‐5A) or with an empty vector (pcDNA3.1) plasmid (MDA‐MB468‐EV). A sample containing rWNT‐5A was included as a control. The insert shows WNT‐5A mRNA levels in MDA‐MB468‐5A and MDA‐MB468‐EV cells. (B) Media from MDA‐MB468‐5A and MDA‐MB468‐EV cells were analyzed by Western blot for content of WNT‐5A protein. A sample containing rWNT‐5A was included as a control. (C) A representative Western blot showing WNT‐5A protein expression in MDA‐MB231 cells stably transfected with the pcDNA3.1(+)‐WNT‐5A plasmid or with an empty vector (pcDNA3.1) plasmid. A sample containing rWNT‐5A was included as a control. The insert shows WNT‐5A mRNA levels in MDA‐MB231 cells stably transfected with the pcDNA3.1 (+)‐WNT‐5A plasmid or with an empty vector (pcDNA3.1) plasmid. The blots are representative of at least four separate experiments. The error bars represent standard error of the mean (n = 4).

Figure S3 Effects of rWNT‐5A, BAPTA, and U0126 on ERK1/2 activity and migration of MDA MB468 cells. (A) ERK1/2 activation was analyzed in MDA MB468 cells either stimulated or unstimulated with rWNT‐5A (0.4 μg/mL) for 30 min, 1 h, 2 h, or 4 h, after which the cells were lyzed in PLB. Quantifications of pERK1/2 in non‐stimulated and rWNT‐5A‐stimulated MDA‐MB468 cells were carried out by calculating integrated density values and normalizing them against total ERK levels. (B) The levels of pERK1/2 were analyzed in MDA MB468 cells in the absence or presence of rWNT‐5A (0.4 μg/mL) for 2, 5, 10, 30 and 60 min. Quantifications of pERK1/2 in non‐stimulated and rWNT‐5A‐stimulated MDA‐MB468 cells were carried out after Western blotting by calculating the integrated density values and normalizing them against total ERK levels. (C) MDA‐MB468 cells were either untreated or incubated with BAPTA/AM (EMD Millipore) at 20 μM for 1 h followed by rWNT‐5A stimulations (for 0, 30 min, or 1 h) in the absence or presence of BAPTA/AM. Quantifications of pERK1/2 in these MDA‐MB468 cells were carried out by calculating integrated density values and normalizing it against total ERK levels. (D) MDA‐MB‐468 cells were treated with 10 μM U0126 (a MEK1/2 inhibitor) for 24h. The cells were then harvested, loaded in the upper chamber of the Trans‐well, and allowed to migrate for 24 h. Cells that had migrated to the bottom of the membrane were counted manually after staining with DAPI. The migration of U0126‐exposed cells was normalized against migration of vehicle‐exposed control cells. Statistical comparisons between means were made with Student's t‐test. The error bar represents standard error of the mean (n = 3). **p < 0.01.

Figure S4 Evaluation of the transient transfections of Cdc42 mutants in MDA‐MB468 cells. Transient transfections of MDA‐MB468 cells with constitutively active Cdc42 (pRK5myc‐Cdc42L61; A) or dominant negative Cdc42 (pRK5myc‐Cdc42N17; B) were performed using the pRK5myc empty vector transfected cells as control (as described in Materials and methods). The lysates were either directly analyzed by Western blot for its content of total Cdc42 and α‐tubulin or used for GST‐PAK1 PBD pull down and subsequent analysis of active Cdc42 (Cdc42‐GTP).

Figure S5 Expression analysis of MMP9 by Immunofluorescence microscopy in MDA‐MB468‐5A cells. MDA‐MB468‐5A and MDA‐MB468‐EV cells growing on cover‐slips were stained with a MMP9 antibody (from Epitomics). For visualization of MMP9 a secondary goat anti‐rabbit Alexa‐488 labeled antibody was used, after which the cells were counterstained with Phalloidin‐TRITC. Arrows show the cytoplasmic localization of MMP9 in the cells. The photomicrographs are representatives of at least three separate experiments.

## Supplementary data 1

Supplementary data related to this article can be found at http://dx.doi.org/10.1016/j.molonc.2013.04.005.
