# Supplementary figures and images for: WNT‐5A triggers Cdc42 activation leading to an ERK1/2 dependent decrease in MMP9 activity and invasive migration of breast cancer cells
Source: Mol Oncol. 2013 Apr 28;7(5):870–83. doi: 10.1016/j.molonc.2013.04.005 (PMC5528454; doi:10.1016/j.molonc.2013.04.005)

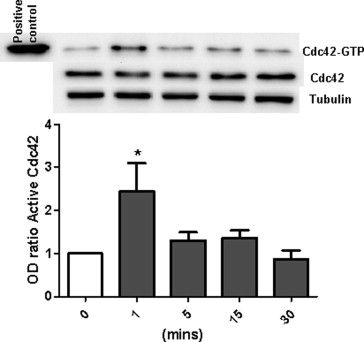

Supplement: Supplementary file 1 — Figure S1 EGF‐mediated activation of Cdc42 in MDA‐MB468. MDA‐MB468 cells were stimulated with EGF(100 ng/ml) for the indicated periods of time (0–30 m). Following these stimulations the cells were lysed and either directly analyzed by Western blotting for its content of total Cdc42 or α‐tubulin or used for GST‐PAK1 PBD pull down and subsequent analysed for the level of active Cdc42 (Cdc42‐GTP). Quantifications of Cdc42‐GTP in EGF stimulated MDA‐MB468 cells were carried out by calculating the Integrated Density Values and normalizing them against total Cdc42 levels. The error bars represent standard error of the mean (n = 3). [file MOL2-7-0870-s001.jpg]

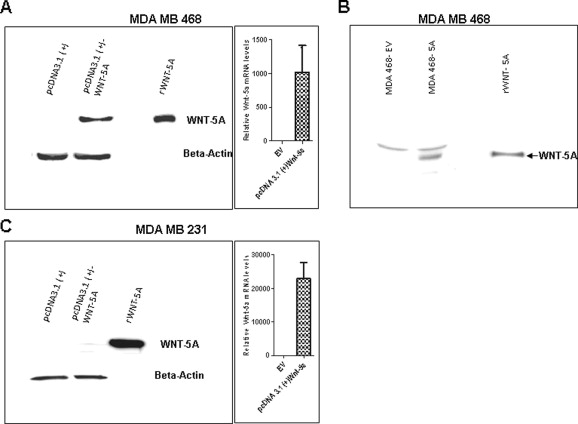

Supplement: Supplementary file 2 — Figure S2 Establishment of breast cancer cells stably expressing the WNT‐5A protein. (A) A representative Western blot showing WNT‐5A protein expression in MDA‐MB468 cells stably transfected with the pcDNA3.1(+)‐WNT‐5A plasmid (MDA‐MB468‐5A) or with an empty vector (pcDNA3.1) plasmid (MDA‐MB468‐EV). A sample containing rWNT‐5A was included as a control. The insert shows WNT‐5A mRNA levels in MDA‐MB468‐5A and MDA‐MB468‐EV cells. (B) Media from MDA‐MB468‐5A and MDA‐MB468‐EV cells were analyzed by Western blot for content of WNT‐5A protein. A sample containing rWNT‐5A was included as a control. (C) A representative Western blot showing WNT‐5A protein expression in MDA‐MB231 cells stably transfected with the pcDNA3.1(+)‐WNT‐5A plasmid or with an empty vector (pcDNA3.1) plasmid. A sample containing rWNT‐5A was included as a control. The insert shows WNT‐5A mRNA levels in MDA‐MB231 cells stably transfected with the pcDNA3.1 (+)‐WNT‐5A plasmid or with an empty vector (pcDNA3.1) plasmid. The blots are representative of at least four separate experiments. The error bars represent standard error of the mean (n = 4). [file MOL2-7-0870-s002.jpg]

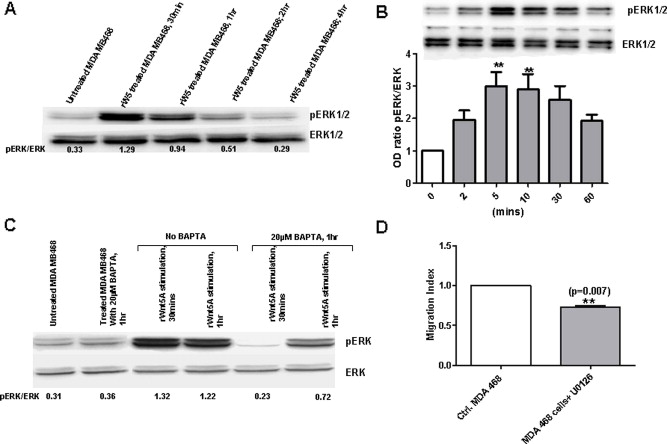

Supplement: Supplementary file 3 — Figure S3 Effects of rWNT‐5A, BAPTA, and U0126 on ERK1/2 activity and migration of MDA MB468 cells. (A) ERK1/2 activation was analyzed in MDA MB468 cells either stimulated or unstimulated with rWNT‐5A (0.4 μg/mL) for 30 min, 1 h, 2 h, or 4 h, after which the cells were lyzed in PLB. Quantifications of pERK1/2 in non‐stimulated and rWNT‐5A‐stimulated MDA‐MB468 cells were carried out by calculating integrated density values and normalizing them against total ERK levels. (B) The levels of pERK1/2 were analyzed in MDA MB468 cells in the absence or presence of rWNT‐5A (0.4 μg/mL) for 2, 5, 10, 30 and 60 min. Quantifications of pERK1/2 in non‐stimulated and rWNT‐5A‐stimulated MDA‐MB468 cells were carried out after Western blotting by calculating the integrated density values and normalizing them against total ERK levels. (C) MDA‐MB468 cells were either untreated or incubated with BAPTA/AM (EMD Millipore) at 20 μM for 1 h followed by rWNT‐5A stimulations (for 0, 30 min, or 1 h) in the absence or presence of BAPTA/AM. Quantifications of pERK1/2 in these MDA‐MB468 cells were carried out by calculating integrated density values and normalizing it against total ERK levels. (D) MDA‐MB‐468 cells were treated with 10 μM U0126 (a MEK1/2 inhibitor) for 24h. The cells were then harvested, loaded in the upper chamber of the Trans‐well, and allowed to migrate for 24 h. Cells that had migrated to the bottom of the membrane were counted manually after staining with DAPI. The migration of U0126‐exposed cells was normalized against migration of vehicle‐exposed control cells. Statistical comparisons between means were made with Student's t‐test. The error bar represents standard error of the mean (n = 3). **p < 0.01. [file MOL2-7-0870-s003.jpg]

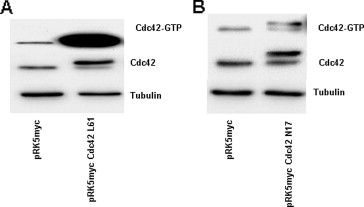

Supplement: Supplementary file 4 — Figure S4 Evaluation of the transient transfections of Cdc42 mutants in MDA‐MB468 cells. Transient transfections of MDA‐MB468 cells with constitutively active Cdc42 (pRK5myc‐Cdc42L61; A) or dominant negative Cdc42 (pRK5myc‐Cdc42N17; B) were performed using the pRK5myc empty vector transfected cells as control (as described in Materials and methods). The lysates were either directly analyzed by Western blot for its content of total Cdc42 and α‐tubulin or used for GST‐PAK1 PBD pull down and subsequent analysis of active Cdc42 (Cdc42‐GTP). [file MOL2-7-0870-s004.jpg]

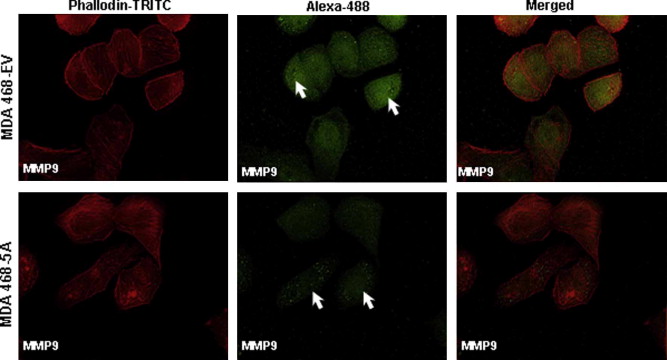

Supplement: Supplementary file 5 — Figure S5 Expression analysis of MMP9 by Immunofluorescence microscopy in MDA‐MB468‐5A cells. MDA‐MB468‐5A and MDA‐MB468‐EV cells growing on cover‐slips were stained with a MMP9 antibody (from Epitomics). For visualization of MMP9 a secondary goat anti‐rabbit Alexa‐488 labeled antibody was used, after which the cells were counterstained with Phalloidin‐TRITC. Arrows show the cytoplasmic localization of MMP9 in the cells. The photomicrographs are representatives of at least three separate experiments. [file MOL2-7-0870-s005.jpg]
